# FUNCTIONAL OUTCOME IN HOME HEALTH AMONG TRADITIONAL MEDICARE AND MEDICARE ADVANTAGE BENEFICIARIES WITH DEMENTIA

**DOI:** 10.1093/geroni/igae098.3572

**Published:** 2024-12-31

**Authors:** Rashmita Basu

**Affiliations:** East Carolina University, Greenville, North Carolina, United States

## Abstract

This study examines the difference in functional outcomes among Traditional Medicare (TM) and Medicare Advantage (MA) beneficiaries with Alzheimer’s disease and related dementias (ADRD) during a home health admission, which is unknown. This cohort study used data from the 2019 Outcome and Assessment Information Set (OASIS) and Medicare claims (Home Health (HH) and inpatient), enrollment, and Chronic Condition Warehouse (CCW) files from the CMS. Functional outcome was measured as a change in the composite ADL score (grooming, bathing, toileting, dressing, walking, transferring, ambulation, and hygiene) from HH admission to discharge. A binary logistic model with an outcome variable (1/0: if the composite score was ≤0 (improvement or static) at discharge (1), and 0 if score >0 (decline) from HH admission to discharge) was estimated. The independent predictor was a binary indicator of MA enrollment (if enrolled ≥1 month in 2019). The ADRD cohort was identified based on CCW criteria and ICD-10 codes from inpatient and HH claims. The analysis included 81,735 Medicare beneficiaries; 30.5% had MA, and 69.5% had TM. There was no difference in the number of HH visits between TM and MA enrollees (2.14 vs. 2.13; P=0.39). Adjusting for patients’ demographics, total number of diagnoses, and availability of caregiving support, MA enrollees were 26% less likely to report improvement or maintenance of ADL composite score (95% CI: 0.71-0.77) than TM. A less favorable outcome for MA enrollees highlights the importance of addressing the inefficient use of home health services among people with ADRD in MA.

